# Downregulation of Talin-1 expression associates with increased proliferation and migration of vascular smooth muscle cells in aortic dissection

**DOI:** 10.1186/s12872-017-0588-0

**Published:** 2017-06-20

**Authors:** Xiaolong Wei, Yudong Sun, Yani Wu, Jiang Zhu, Bin Gao, Han Yan, Zhiqing Zhao, Jian Zhou, Zaiping Jing

**Affiliations:** 1 Department of Vascular Surgery, Changhai Hospital, Second Military Medical University, 168 Changhai Road, Shanghai, 200433 China; 2 Company 8, Cadet brigade, Second Military Medical University, Shanghai, China

**Keywords:** Talin-1, Aortic dissection, Vascular smooth muscle cell, Proliferation, Migration

## Abstract

**Background:**

This study aimed to assessed whether Talin-1 is involved in the pathogenesis of aortic dissection via regulating vascular smooth muscle cell (VSMC) biological function.

**Methods:**

Human aortic samples were obtained from organ donors who died from nonvascular diseases as normal controls and from patients undergoing surgical repair of thoracic aortic dissection. The expression level and distribution of Talin-1 were detected using westernblot analysis and immunohistochemistry in each sample. We inhibited the expression of Talin-1 via RNA interference in VSMCs. VSMC proliferation was detected by Cell-counting Kit-8 analyses. Scratch test and flow cytometry were used to identify the migration and apoptosis ability. Antibody microarray analysis and qRT-PCR were used to detect some protein and mRNA changes which were induced by Talin-1 downregulation.

**Results:**

Talin-1 was significantly downregulated in the media of aortic dissection samples compared with controls (*P* < 0.05). Talin-1 knockdown significantly induced VSMC proliferation and migration in vitro. Proteins which involved in cell cycle can be regulated by downregulating Talin-1. Down regulation of Talin-1 can significanly increased the expression of anaphase-promoting complex subunit 2 (APC2) and decreased p19 alternative reading frame (p19ARF), Cullin-3, and beta actin’s expression.

**Conclusions:**

Talin-1 induces VSMCs proliferation and migration. It downregulated in aortic dissection, which might play a potential role in the development of aortic dissection.

## Background

Aortic dissection (AD) is one of the catastrophic medical illness and it may be rapidly fatal without early diagnosis and appropriate management [1]. Since in-depth cognition on AD’s pathological process can improve its clinical treatment strategy, it is important to further clarify the mechanisms of AD. Pathological vascular remodeling plays a key role in AD [2, 3]. As the major cell in aortic media, the functional change of vascular smooth muscle cell (VSMC) has been thought critical for maintaining the normal biomechanical properties of the aortic wall [4–6]. Dysfunctions of proliferation and migration in VSMC has been reported to participate in vascular diseases [7, 8]. Although the pathogenesis of AD has been studied in recent years, it still has not been thoroughly elucidated.

Talin-1 is expressed in nearly every tissue of vertebrates [9]. Talin-1 located at the adhesion complex between cells and their extracellular matrix (ECM) and it regulates integrin and focal adhesion signaling [10, 11]. Major insights into the function of Talin-1 were derived from experiments demonstrating that the Talin-1 head binds the cytoplasmic tails of β integrin subunits, leading to the final step in “inside-out” integrin activation [12, 13]. Talin-1 functions as an adaptor protein and further promoting integrin-mediated signal transduction [14]. Recent studies revealed that the dysregulation of Talin-1 can lead to disease states in cell spreading, migration, and survival which has led to extensive investigation into its role in cancer and hematologic disorders [15–18]. However, the relationship between Talin-1 and aortic disease has never been reported.

In this study, we demonstrate the expression characteristics of Talin-1 in aortic specimens of AD, and investigated Talin-1’s effects on VSMCs’ function. Thus, we sought to determine some proteins involved in the process, to observe the relationship between Talin-1 expression and the proliferation and migration of VSMC, and to determine the relationship between Talin-1 expression and AD.

## Methods

### Patient recruitment and sample collection

Aortic fragments were taken from ten patients who underwent surgical repair of thoracic aortic dissection. The maximum aorta diameter of these patients was <5.0 cm. Patients with Ehlers-Danlos syndrome, bicuspid aortic valves, Marfan syndrome, aortic aneurysm, other connective tissue disorders and traumatic aortic injuries were excluded. Control aortic tissues were obtained from ten organ donors who died from nonvascular diseases. Aortic tissue of every specimen was collected within 30 min after aorta excision. All of the specimens were rinsed at least five times with precooled saline solution to remove the blood and mural thrombus adhering to the vascular wall. We clipped a small piece of arterial wall near tearing dissection and removed adventitia and thrombus immediately using sterile tweezers and eye scissors in a clean Petri dish. All AD and normal aorta (NA) group tissue samples were divided into two parts. One part was cut into approximately 2-mm and placed it in sterile EP tubes. The specimens were then rapidly frozen in liquid nitrogen and stored at 80 °C until use. The entire procedure was completed in 10 min. The second part was fixed in 4% paraformaldehyde and then embedded in paraffin. We did not perform additional surgery beyond standard of care required to obtain these additional specimens. The demographic and clinical characteristics of the included patients and controls are presented in the Table 1. The study was conducted following the principles outlined in the Declaration of Helsinki and approved by the Changhai Hospital medical ethic committee. Informed consent was obtained from each subject in AD group and entitled relatives in control group.

**Table 1:** The demographic and clinical characteristics of the included patients and controls

	Patients (*n* = 10)	Controls (*n* = 10)	*P* value
Age, years	46.84 ± 5.31	47.20 ± 6.56	0.89
Sex, male:female	10:0	10:0	1.0
Hypertension, n(%)	8 (80.00%)	2 (20.00%)	<0.01
Hyperlipidemia, n(%)	1 (10.00%)	0 (0.00%)	0.31
Diabetes mellitus, n(%)	0 (0.00%)	0 (0.00%)	1.0
Smoking history, n(%)	4 (40.00%)	5 (50.00%)	0.65
Stanford classification, n(%)			-
Type A	10 (100.00%)	-	
Type B	0 (0.00%)	-	

### Western blotting

Total protein was extracted by 1000 mL of RIPA buffer from 100 mg tissue. For cells, VSMCs were washed 3 times in PBS and 100 μL RIPA buffer (Cell Signaling Technology, MA, USA) per well (6-well plate) applied to cells. Cell lifter (Corning Costar) was used to mix and lyse the cells, which were subsequently transferred to eppendorf tubes. All of the samples were standardized to 1.0 mg/mL. A total of 20 mL was loaded on a 6% to 12% sodium dodecyl sulfate polyacrylamide gel electrophoresis plate and transferred onto a polyvinylidenedifluoride membrane, so the total amount of each sample of one western blot gel was 20 μg. Membranes were blocked with 5% nonfat milk (Becton Dickinson, Franklin Lakes, NJ), washed, and probed with the following primary antibodies: rat anti-human Talin (1:1000; Abcam, Cambridge, United Kingdom), and GAPDH (1:500; Abcam). After washing, membranes were incubated with a horseradish peroxidase-conjugated goat anti-rat (1:2000; Sigma) for 1 h. Then, membranes were washed and developed using an enhanced chemiluminescence kit according to the manufacturer’s instructions (Thermo Fisher). Western blot assays were repeated three times. After scanning the blots using a flatbed scanner, the band intensities were analyzed using the Image-ProPlus software version 6.0 (Media Cybernetics, Inc., Rockville, MD).

### Immunohistochemistry

Immunohistochemistry staining for Talin-1 was performed on 5 um thick paraffin sections from human aorta samples which were mentioned in “Patient recruitment and sample collection”. Maxvision TM system (MAXIM) was used to perform immunohistochemical. Five paraffin-embedded sections of each groups were routinely hydrated and then microwave treated for antigen restoration. 3% hydrogen peroxide was used to inactive endogenous peroxidase for 15 min at room temperature. Non-immune rat serum was used to block the sections blocked for 10 min. Then, rat anti-human Talin-1 (1:500, Abcam) was used to incubate for 1 h at room temperature. Maxvision TM was applied to each slide and then incubated for 15 min at room temperature. DAB staining was perpormed with mixed liquid A, B, C (Maxvision TM kit) in same volume. All these sections were counterstained with hematoxylin, cleared, mounted, and observed under a light microscope. Negative controls were carried out replacing the primary antibody with non-immune rabbit serum. Semi-quantitative analyses of positive signals in samples were performed by Image-ProPlus software version 6.0.

### Cell culture and transfection

Prime VSMCs from human aorta were purchased from Sciencell and cultured in mixed culture media (M199, GIBCO/15% FBS, GIBCO/2% A-A, GIBCO) at 37 °C in a humidified atmosphere with 5% CO^2^. All of the experiments were performed with cells in the third to sixth passages. VSMCs were maintained in serum free M199 for 12 h before each treatment. Talin-1 lentivirus and vector harboring green fluorescent protein and the control adenovirus (green fluorescent protein adenovirus) were constructed by Obio (Shanghai, China). The siRNA sequences targeting Talin-1 were as follows: 5′-GCTCGAGATGGCAAGCTTA-3′. The sequences of control shRNA were as follows: 5′- TTCTCCGAACGTGTCACGT-3′. The adenovirus for the knockdown of Talin-1 were provided by Obio. The adenovirus was diluted in 0.2 mL complete medium at 10^8^ transduction units per mL containing hexadimethrine bromide (Polybrene; 8 mg/mL) and were incubated with the cells with a multiplicity of infection of 60 for 24 h at 37 °C. Next, the medium was then replaced with M199 and the cells were cultured for 48 h.

### Cell proliferation assay

Cell proliferation was detected by Cell-counting Kit-8 (Dojindo) according to the manufacturer’s instructions. VSMCs were seeded in 96-well plates at a density of 5 × 10^3^ cells per well. After cell adhesion, VSMCs were incubated with 1% FBS M199 media for 24 h. The VSMCs were then treated with adenovirus at the multiplicity of infection of 120 with 15% FBS M199. The medium was replaced every 24 h. At 0, 12, 24, 36 and 48 h after transfection, 10 mL of Cell-counting Kit-8 reagent was added to each well, and the cells were further incubated for 4 h at 37 °C. Spectrophotometric plate reader was used to read the absorbance at 450 nm. The negative controls were VSMCs that had been transfected by adenovirus without the Talin-1 interfering effect.

### Scratch test

VSMCs were resuspended in complete medium, with density adjusted to 1 × 10^6^ cells/ml. Then, 2 ml of cell suspension was added to each well of a 6-well plate. At 80% confluence, the medium was removed, a straight-line scratch was made on a monolayer of cells using a standard 1000 μl plastic pipette tip 48 h after transfection. The cells were washed using Dulbecco’s Phosphate Buffered Saline (DPBS). For each well, pictures were taken at 0 and 24 h after scratch. The migration cell numbers was calculated at the 2 time points.

### Apoptosis assay by flow cytometry

The cells were washed twice with DPBS and resuspended in 1X binding buffer (KeyGEN BioTECH, Nanjing, China) at a concentration of 1 × 10^6^ cells/ml. Cells were stained with Annexin V-APC and 7-amino-actinomycin D (7-AAD), using the Annexin V apoptosis detection kit (KeyGen Biotech) and detected by flow cytometry. The experiments were repeated at least three times.

### Antibody microarray analysis

Antibody Microarray (ACC058) was obtained from Full Moon BioSystems (Sunnyvale, CA, USA). Each glass slide contains 62 highly specific and well-characterized antibodies in duplicate. These antibodies play important roles in diverse biological functions such as cell proliferation and migration which were raised against cellular proteins. Proteins were extracted as described above, biotinylated and hybridized to the microarray. Antibody Microarray Detection Kit (Spring Biosci- ence, Pleasanton, CA, USA) was used to detecte the signal with fluorescent-labeled strepatavidin according to the manufacturer’s protocol. A change of around 1.5-fold was used as a cut-off standard to evaluate the differential expression of proteins between anti-Talin-1 treated and anti-GFP treated VSMCs.

### RNA isolation and quantitative reverse transcription-polymerase chain reaction (qRT-PCR)

Total RNA was extracted from the cultured cells in accordance with the manufacturer’s instruction for the miRNease Mini Kit (Qiagen, Hilden, Germany). PrimeScript RT reagent Kit (TaKaRa, Tokyo, Japan) was used to synthesize complementary DNA with 500 ng of total RNA. According to gene sequences published in GenBank, primers showed in Table 2 were designed using Primer 5.0 software and they were checked by oligo 7 and NCBI Blast. qRT-PCR analyses were performed using LightCycler 480 SYBR Green I Master (Roche, Welwyn Garden, Swiss). A negative control (without template) was always included in each qRT-PCR step. Each SYBR Green reaction (total volume, 20ul) contained 2 ul of cDNA as template and each primer at 0.25 um. We incubated the reactions at 95 °C for 30 s followed by 40 cycles at 95 °C for 5 s, at 55 °C for 10 s and at 72 °C for 15 s. To verify that the SYBR Green dye detected only one qRT-PCR product, we subjected the samples to the heat dissociation protocol after the final cycle of qRT-PCR to check for the presence of only peak. With GAPDH as internal controls, the reliability of qRT-PCR was evaluated by the melting curve. The (cycle threshold) Ct value (power amplification knee point) was gotten, and the relative expressions of target genes were calculated by 2^-△△^Ct method. All experiments in qRT-PCR technique included at least three replicates per group.

**Table 2:** The sequence of primers in this study

	Sequence (5′ to 3′)
APC2	Forward: TACCGCCGTGCCATGAACGA Reverse: CCGGGTCATCTTGTGCATCTCA
P19ARF	Forward: ACCCACCCCGCTTTCGTA Reverse: GCTCACTCCAGAAAACTCCAAC
GSK3B	Forward: AGTCCGATTGCGTTATTTCTTC Reverse: ACAGGGAGCGTCTGTTTGG
ROCK1	Forward: TGGTGCTGGTAAGAGGGCATT Reverse: CCGCAGCAGGTTGTCCATT
ACTB	Forward: GTCCACCGCAAATGCTTCTA Reverse: TGCTGTCACCTTCACCGTTC
CUL3	Forward: AGTCGTAGACAGAGGCGCAATA Reverse: CCGTTGATTTGTCAAGGCAGT
GAPDH	Forward: AATCCCATCACCATCTTCCAG Reverse: GAGCCCCAGCCTTCTCCAT

### Statistical analysis

Data are presented as mean ± SD. Experimental data were analyzed using the Statistical Package for the Social Sciences (SPSS) 17.0 software (SPSS Inc., Chicago, USA). Groups were compared using independent samples *t* test and one-way analysis of variance (ANOVA). *P* < 0.05 was considered statistically different.

## Results

### Differential expression of Talin-1 protein in aortic dissection versus normal aorta tissue specimens

**Fig. 1:**
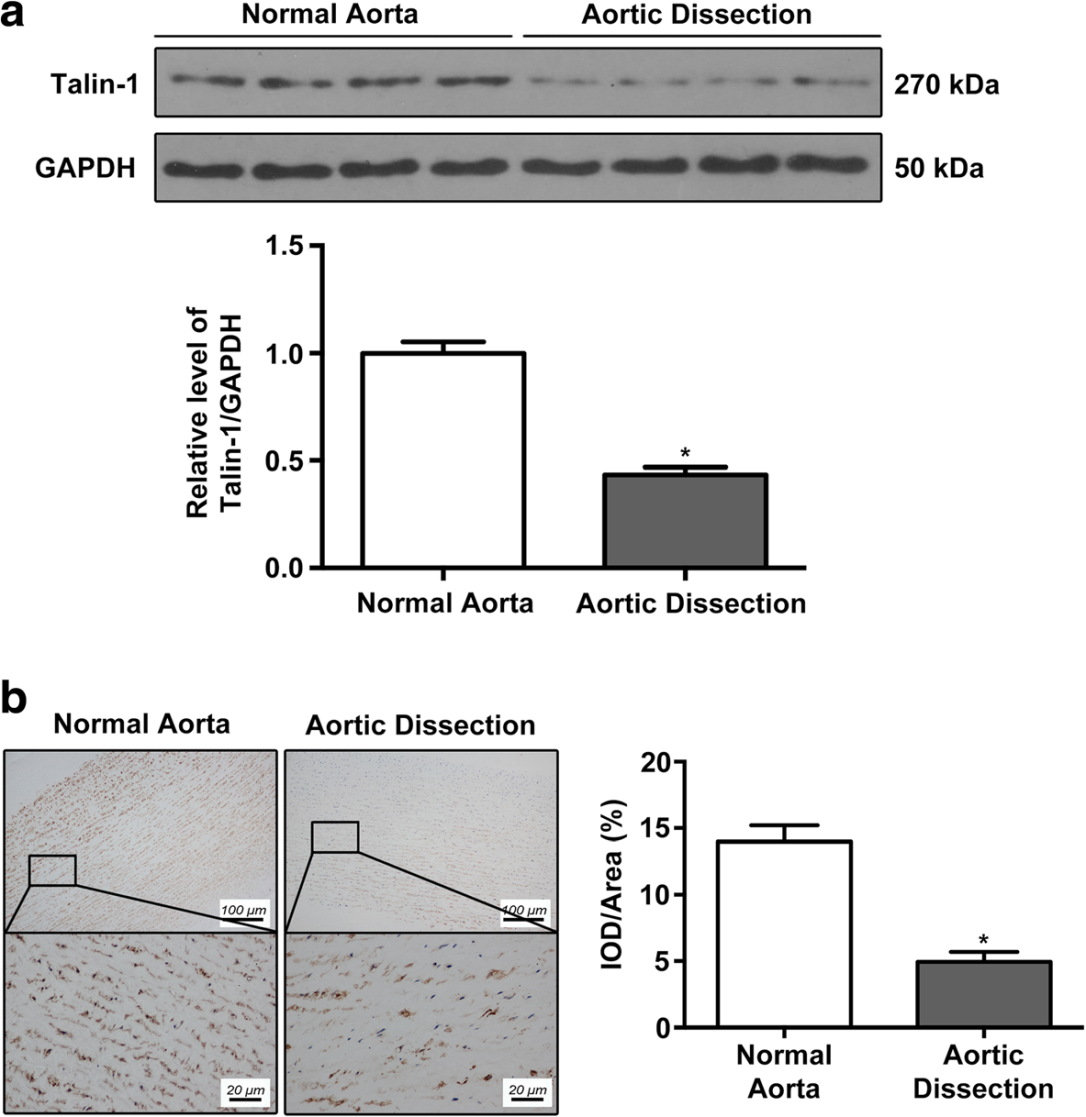
Talin-1 is downregulated in the media of human aorta samples with aortic dissection. **a** Western blot for Talin-1 expression in human aorta samples (*n* = 10 per group). Talin-1 was significantly downregulated in AD samples (*P* < 0.01). **b** Immunohistochemistry on the same sections of human aorta with Talin-1 staining (*n* = 10 per group). Quantitative analysis of the results of immunohistochemistry demonstrated that Talin-1 was distributed in the media of aorta where VSMCs were located (*P* < 0.01). Data are represented as mean ± SD

To reveal the expression features of Talin-1 in human aorta, we use western bot analysis to detect the expression of Talin-1 in AD and their matched NA tissues (Fig. 1a). Talin-1 was significantly lower in the AD group. Quantitative analysis of western blot results confirmed the differences between the two groups. Then, to further detect the distribution of Talin-1 in human aorta tissues, immunohistochemistry was performed. Immunohistochemical staining with Talin-1 antibodies was done on cross-sections of the straight part of aorta. As shown in Fig. 1b, Talin-1 was decreased in the AD tissues compared with the NA tissues (*P*<0.05) and it was mainly distributed in the media of human aorta tissues. All of these results suggest that Talin-1 showed a downregulation character in AD tissues especially in the media of the aortic wall compared with NA tissues.

### Vascular smooth muscle cells’ proliferation and migration were increased by negative regulating Talin-1

**Fig. 2:**
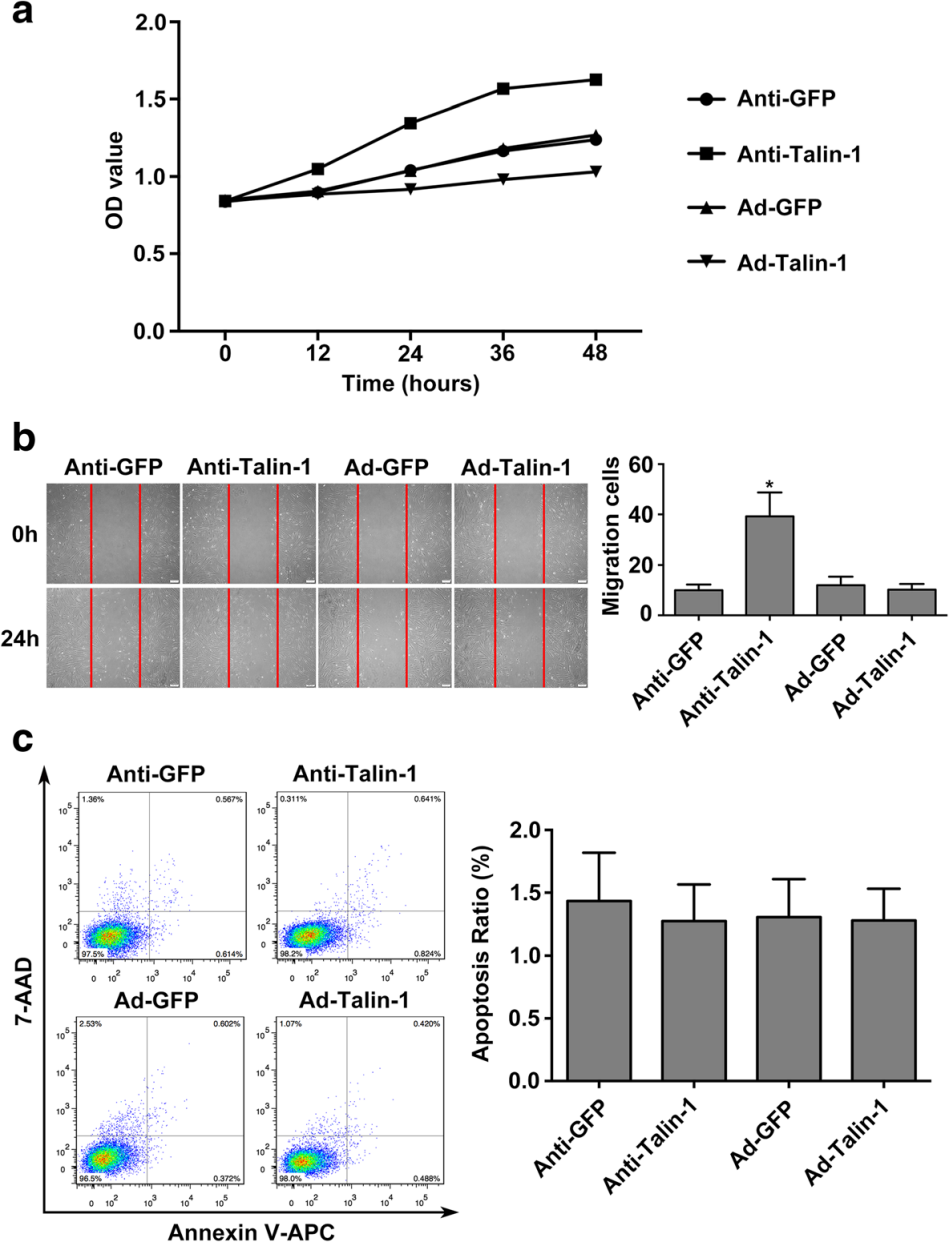
In vitro inhibition of Talin-1 strongly promotes VSMCs’ proliferation and migration. **a** Proliferation curves of VSMCs among Ad-Talin-1, Ad-GFP, Anti-Talin-1 and Anti-GFP groups. The result showed that the proliferation ability of VSMCs was significanly improved at 24 h after the treatment of adenovirus in Anti-Talin-1 group. In the contrary, Ad-Talin-1 group inhibited the proliferation ability of VSMCs when compare with Ad-GFP group (*n* = 5 per group). **b** Results of the scratch test showed that migration capability of VSMCs was improved in Anti-Talin-1 group. However, Ad-Talin-1 group didn’t significantly decreased the migration capability of VSMCs. Quantified data were presented as the number of migration cells per high-power field (*n* = 5 per group). **c** Flow cytometer analysis showed that knockdown of Talin-1 had no effect on VSMCs’ apoptosis (*n* = 5 per group). Data are represented as mean ± SD. All the experiments were performed thrice

To investigate whether the Talin-1 plays a relevant role in the regulation of VSMC biological function, we transfected the VSMCs with adenovirus to interfere the expression of Talin-1. As determined by the growth curve and the CCK-8 assay, downregulation of Talin-1 in VSMCs caused a significant increase in the amount of proliferating cells (Fig. 2a). However, overexpression of Talin-1 can significantly decrease the VSMCs’ proliferation. To further confirm the biological role of Talin-1 on VSMC, we performed scratch test to detect VSMCs’ migration function. Treatment with the Talin-1 inhibitor adenovirus was sufficient to substantially increase the migration response in cultured VSMCs (Fig. 2b). In contrast, there was no effect on migration when upregulated Talin-1’s expression. Furthermore, to evaluate the effect of Talin-1 on VSMC apoptosis, we performed a flow cytometry test. There was no significant change in the apoptosis of VSMCs after transfection with adenovirus to interfere Talin-1’s expression (Fig. 2c). These in vitro observations reveal that the Talin-1 is a critical regulator of VSMC proliferation and migration.

### Association of cell cycle related proteins and Talin-1 expression with functional change of vascular smooth muscle cells

**Fig. 3:**
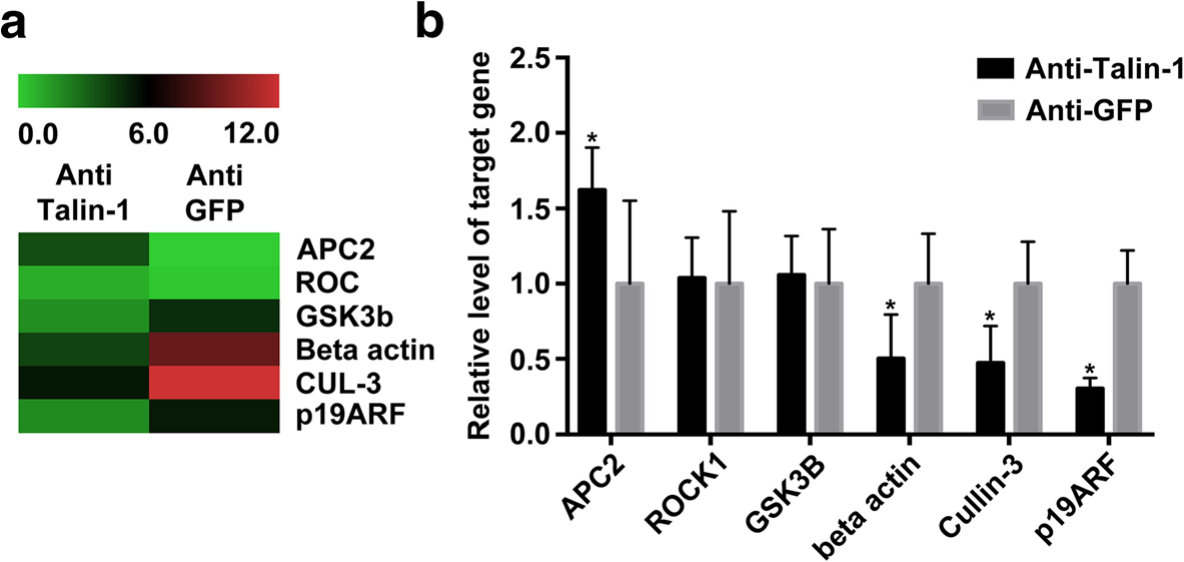
Proteins related with cells’ proliferation has differential expression in VSMCs with Talin-1 downregulated. **a** Cluster analysis of protein expression in a 62 proteins antibody microarray which measured from VSMCs with Talin-1 downregulated. There were six proteins showed obviously changed when compared with Anti-GFP group. **b** Analysis using qRT-PCR showed that APC2 mRNA levels were significantly higher in Anti-Talin-1 group. In the contrary, p19ARF, Cullin-3, and beta actin were significant lower in Anti-Talin-1 group when compared with Anti-GFP group. Data are represented as mean ± SD

To identify proteins related with cell cycle that are altered during the downregulation of Talin-1 in VSMCs, we compared protein expression levels in anti-Talin-1 and anti-GFP by antibody microarray analysis. The results revealed that standing out from 62 proteins measured, there were six significantly changed proteins (Fig. 3a, change around 1.5-fold). An independent qRT-PCR assay confirmed the significant increased expression of Anaphase-promoting complex subunit 2 (APC2) and the decreased expression of p19 alternative reading frame (p19ARF), Cullin-3, and beta actin (Fig. 3b).

## Discussion

In this study we sought to find the expression pattern of Talin-1 in AD and its regulatory role in VSMCs’ biological function. We identified that Talin-1 mainly expressed in the media of human aortic samples and significant downregulation of Talin-1 was found in AD aortic samples. The weakening of the aortic wall induced by abnormal function of VSMCs has been thought as the initial step of pathologic remodeling in AD. We used the adenovirus technology to knockdown Talin-1 expression in VSMCs. As shown above, Talin-1 knockdown VSMCs showed increased proliferation and migration, confirming the involvement of Talin-1 in AD pathological process. The expression of proteins involved in cell cycle such as APC2, p19ARF, Cullin-3 and beta actin were changed during this process. In addition, further experiments to reveal whether the expressive discrepancy of Talin-1 exists in other vascular diseases such as aortic aneurysm will be a focus of future study.

Recent researches have shown that AD is featured by increased collagen deposition, elastin fragmentation and inflammation infiltration [19–21]. Our previous reported that 126 differentially expressed proteins, especially some cytoskeletal proteins, were found between the AD and normal aorta tissue using two-dimensional gel electrophoresis [22, 23]. Some proteins were thought as a therapeutic target in the treatment of AD [24, 25]. Talin-1 is a large focal adhesion protein that links intracellular networks with the extracellular matrix (ECM) through its connection with the membrane integrins and actin cytoskeleton [26]. A properly structured ECM determines the mechanical integrity of the aorta wall. Elastin and collagen are key structural components of the ECM, contributing to the stability and elasticity of normal arteries. It has been reported that Talin-1 related with elastic and collagen’s expression [27–29]. Although recent studies have confirmed a role for the dysregulation of Talin-1 in some diseases [30, 9]. Its role in aortic disease has not been fully illuminated. Our study found that Talin-1 was downregulated in the medial part of the aortic wall in AD. Thus Talin-1 might participate in the development of AD’s pathologic process.

Early investigations have reported VSMCs played a critical role in aortic wall reconstruction which participated in AD pathological process [31–33]. Thus, we attempt to evaluate Talin-1’s regulatory role in VSMCs’ biological function. It has been confirmed that abnormal proliferation of VSMCs might be the main cause of pathologic vascular remodeling and vascular disease occurrence via affecting the stability of vascular media structure and function [34]. Müller BT, et al. and Wang L, et al. have reported that VSMCs from AD tissues seem to proliferate faster than normal aorta tissues and the genes that participate in proliferation showed an elevated level of expression [35, 36]. Furthermore, VSMC migration is also acknowledged as a crucial issue in AD pathogenesis [37, 38]. Our vitro functional experiments verified that downregulation of Talin-1 drastically enhanced the proliferation and migration capability of VSMCs which implied the occurrence of a positive pathological remodeling that was harmful to AD development. Extensive study of proteins involved in cell cycle has been conducted via the antibody microarray analysis. APC2 was significantly increased when inhibited Talin-1’s expression. Wen H et al. found that when free APC2 was decreased the proliferation of cell was inhibited [39]. Our in vitro study found that the VSMCs proliferation was promoted when Talin-1 was decreased and APC2 might play an important role in this process. Cullin-3, p19ARF and beta actin were significantly downregulated when inhibited Talin-1’s expression. They have been confirmed participated in regulating cell’s proliferation [40–42]. Some studies found that Cullin-3 and beta actin regulated cell migration [43, 44]. The relationship between these proteins and Talin-1 has not been researched.. These results have provided the potential targets within the regulation effect of Talin-1 on VSMCs proliferation and migration, as well as on the processes of AD’s development. Our further research will focus on clarifying the mechanisms of Talin-1’s regulatory role on VSMC’s biological function and further provided new insight in researching the biological behavior of Talin-1, as potential therapeutic agent for treatment of AD.

## Conclusions

In summary, the present study identified AD tissue-specific expression of Talin-1 in detail for the first time. Combined with HASMC function, our founding suggested Talin-1’s role in regulating VSMC proliferation and migration and further identified some proteins which might related with this process. Overall, our results will be conducive to illuminate the pathologic mechanism in AD. Future studies are required to identify the effect of Talin-1 on AD pathogenesis, and to explore the detailed molecular mechanism by regulating proliferation and migration of VSMCs.

## Abbreviations

7-AAD: 7-amino-actinomycin D
AD: aortic dissection
APC2: anaphase-promoting complex subunit 2
DPBS: Dulbecco’s Phosphate Buffered Saline
ECM: extracellular matrix
NA: normal aorta
p19ARF: p19 alternative reading frame
qRT-PCR: quantitative reverse transcription-polymerase chain reaction
VSMC: vascular smooth muscle cell
